# Impact of the Relative Age Effect on Competition Performance in Basketball: A Qualitative Systematic Review

**DOI:** 10.3390/ijerph17228596

**Published:** 2020-11-19

**Authors:** Alfonso de la Rubia Riaza, Jorge Lorenzo Calvo, Daniel Mon-López, Alberto Lorenzo

**Affiliations:** Facultad de Ciencias de la Actividad Física y del Deporte (INEF-Departamento de Deportes), Universidad Politécnica de Madrid, C/ Martín Fierro, 7, 28040 Madrid, Spain; alfonso.delarubia@upm.es (A.d.l.R.R.); alberto.lorenzo@upm.es (A.L.)

**Keywords:** relative age effect, birthdate, performance, competition, sport talent, sport success, evaluation, statistical, team sport, basketball

## Abstract

Performance in basketball is multifactorial. One of the modifying factors is the “Relative Age Effect—RAE”. However, its impact depends on the sample characteristics and sport context. The purpose of this study was to examine the influence of the RAE on basketball competition performance by analysing peer-reviewed articles published until July 2020. According to the Preferred Reporting Items for Systematic Reviews and Meta-Analyses systematic search guidelines, nine studies were identified in four databases: Sport Discus, PubMed, Web of Science, and Scopus. Moreover, a study quality analysis using “Strengthening the Reporting of Observational Studies in Epidemiology” guidelines was carried out. The results confirmed an impact of the RAE on competition performance in basketball (56% measurements) and a higher influence of the RAE on short-term collective performance (54% measurements). Statistical parameters were affected, especially in men and U14-U18 categories. No impact of the RAE reversal and no influence of the RAE on long-term collective performance were found. There was a higher impact of the RAE in men (71%), the U14-U18 categories (44%), and at the national level (40%) was identified. The RAE has a variable influence on basketball performance according to developmental constraints. Nevertheless, the findings should be considered based on the sport context due to the heterogeneity and variability of the identified results.

## 1. Introduction

The analysis of certain indicators (i.e., technical, tactical, biomechanical, and physiological) is a widely used procedure in the field of sport to recognise qualitatively individual and collective excellence and performance [1,2]. Team sports often use clear, unequivocal, and useful indicators associated with the successful game actions and/or the matches played [3]. By analysing these types of parameters, either in isolation or in comparison with other athletes or teams, an accurate measure of sport success can be obtained through indexing performance in team sports. In basketball, the analysis of sports success can be approached from many perspectives (product/process, victories/defeats, etc.). One perspective is the individual and collective performance analysis: (a) Individual. Using statistical parameters to measure short-term competition performance (i.e., games played, points scored, and average performance indexes) [4,5,6,7] or recognizing personal achievements throughout the player’s sport career (i.e., accumulated statistics, awards, or wages) [8,9]; (b) Collective. Considering the final team classification in one competition [10,11] or success reached by teams (rankings) for periods longer than one season [12,13].

Competition performance in team sports seems to be influenced by, among other factors, the “relative age effect—RAE” [14,15]. Grouping players by competitive levels, according to the chronological age and based on a pre-established cut-off date (usually 1 January), could cause differences that would affect sport performance [16]. Consequently, players born in the first months of the year could find themselves over-represented, having greater possibilities of reaching top professional sport levels and, therefore, achieving higher levels of sport success, both individually and collectively [17,18].

The RAE is a phenomenon normally reflected in youth categories. Thus, the relatively older athletes usually have more opportunities to achieve a higher sport level than their relatively younger counter-peers [19]. Researches have provided different explanations in this regard: Socio-cultural [20], geographic [21], psychological [22] criteria or those linked to the competition itself [23]. However, a biological factor associated with the “maturation-selection hypothesis” is the most cited argument [24,25]. This theory highlights the differences caused by the RAE among players in youth categories, especially in sports associated with a predominantly physical nature [26]. In addition, a different maturational status, due to the almost 12-month development process, would allow relatively older players to enjoy better physical, anthropometric and physiological qualities in compliance with the games’ demands. Consequently, older players may outperform their young sport peers [27].

Nevertheless, in senior categories or top competitive levels, it does not seem as clear that relatively older athletes enjoy certain sport and competitive advantages over their younger peers [28]. Specifically in team sports, the impact of the RAE tends to decrease as the athlete progresses in their sport career [29] but does not disappear completely [30]. Even in professional competitions of the highest level, the trend is usually reversed [31,32,33,34], confirming the “underdog effect” [35]. This phenomenon would be based on an over-representation of relatively young athletes at top senior competition levels. This fact, which is contrary to the RAE, is called “relative age effect reversal” (RAE reversal) [25].

With regard to basketball, some predictive biological success factors, such as height or years at peak of high velocity (“YAPHV”) [36,37], enhance the relationship between the RAE and competition performance. This circumstance conditions the sporting reality in aspects such as the player selection process [38] or the allocation of game positions [39]. This perspective does not seem to consider the athlete’s maturity status, which would cause an imbalance between the athlete’s maturity development and his or her chronological age [27]. Furthermore, due to the different performance profile between positions—guard, forward and center—[40] or, as currently occurs in the National Basketball Association (NBA) or the National Collegiate Athletic Association (NCAA), between the roles of the players depending on the tactical conception of the coach/staff [41], coaches tend to select the players with the greatest biological advantage according to the players’ current performance characteristics (physiological, physical, and/or anthropometric) [42]. However, it is not known how much this strategy affects the individual performance of the player or the team’s collective.

Surprisingly, the RAE has been studied from diverse approaches but, to the best of our knowledge, the impact of the RAE on competition performance in basketball at regional, national and international competition levels, measured both individually and collectively as well as in the short and long-term, has not been studied in-depth. Therefore, the purpose of this study is to conduct a systematic review to determine the influence on competition performance in basketball. We examined the scientific information published between January 2000 and July 2020 to analyze the impact of the RAE on competition performance according to: (a) The performance measurement indicators employed (type of result in competition and performance production period), (b) the sample characteristics (gender and age group), and (c) the sport context (competition category and competition level).

## 2. Materials and Methods

The objective of the present research was to qualitatively synthesize the scientific evidence with regard to the RAE impact on competition performance in basketball. The stages of the review procedure and subsequent analysis of the original articles stayed within to the guidelines set out in the Preferred Reporting Items for Systematic Reviews and Meta-Analysis (PRISMA) checklist and the Population, Interventions, Comparisons, Outcomes, and Study Design (PICOS) question model for the definition of inclusion criteria.

### 2.1. Study Selection and Eligibility Criteria

Primary and original studies for the purpose of evaluating the association between the RAE and competition performance in basketball were included. Furthermore, studies had to have been published in English or Spanish language, in peer-reviewed journals with an impact factor included in the Journal Citation Reports of the Web of Science (JCR of WoS) or Scimago Journal and Country Rank (SJR of Scopus) and in the period January 2000–July 2020 (previously, no significant relevant studies were found).

According to the “PICOS” question model, the inclusion criteria were: (1) “Population”: Basketball players over the age of 13 years-old (minimum age with official competition performance statistics in basketball) with highest standard of performance who participate in the 1st or 2nd competition levels (professional basketball leagues or tournaments at international or national) or 3rd competition level (leagues or tournaments involved in talent identification and development systems) [43]; (2) “Intervention”: Local/regional, national and international official high-performance competitions with statistics about individual and/or collective competition performance; (3) “Comparison”: Association between individual and/or collective competition performance and player’s birthdate; (4) “Outcomes”: Competition performance according to two specific indicators, “type of result” (individual and/or collective) and “performance period” (short term and/or long term); (5) “Study Design”: Observational-descriptive research based on a relationship between the RAE and competition performance in basketball.

The exclusion criteria were: (1) Analyzed the impact of the RAE in other contexts (educational, recreational, fitness, etc.); (2) examined the RAE in individual sports, in pairs or connected to refereeing; (3) examined the RAE in other team sports (i.e., football, handball, ice hockey); (4) not provided data associated with the sample distribution according to the RAE; (5) birthdate distribution no reported or reported by year (even-odd year); (6) exclusively evaluated other different results (acquisition skills, fitness, psychological, physical and/or anthropometric tests); (7) exclusively found a correlation between the RAE and performance in other terms (market value, wage, etc.); (8) examined relationships with other developmental and/or behavioural processes (leadership, anxiety, suicide, etc.); (9) analyzed possible interventions to minimize or eliminate the consequences of the RAE. Systematic reviews in relation to the analysis of the RAE in the sport field were only considered as support material in the search process for potentially valid research and in accordance with the aim of the study.

### 2.2. Search Strategy

Four electronic bibliographic databases were used: Sport Discus, PubMed, Web of Science, and Scopus. The predefined search strategy was carried out using terms grouped into three search strings: (1) “RAE” OR “relative age” OR “relative age effect*” OR “influence of age” OR “birthdate” OR “birthdate effect*” OR “age effect*” OR “season of birth”; AND (2) “basketball” OR “team sport*” OR “professional sport*” OR “elite sport*” OR “associative sport*” OR “collective sport*”; AND (3) “performance” OR “minute* played” OR “game* played” OR “point*” OR “rebound*” OR “goal attempt*” OR “percentage of effectiveness” OR “assist*” OR “turnover*” OR “steal*” OR “blocked shot*” OR “personal foul* committed” OR “performance index rating” “ranking” OR “classification” or “place*” OR “medal*” OR “success” or “attainment” OR “statistics”.

### 2.3. Systematic Review Protocol

**Figure 1:**
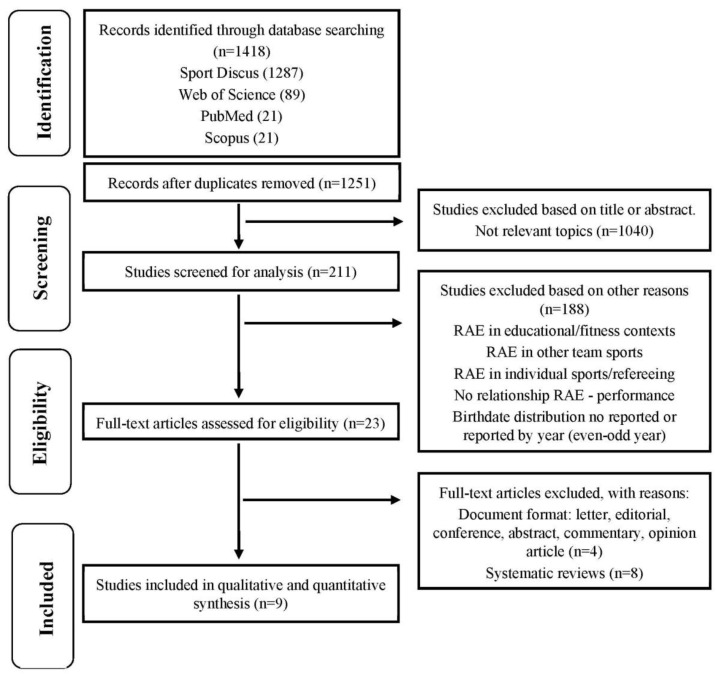
Flow diagram for screening and selection of studies according to Preferred Reporting Items for Systematic Reviews and Meta-Analysis (PRISMA).

The authors worked separately and independently to ensure the reliability of the process and the suitable eligibility of the studies. According to the criteria for preparing systematic reviews “Preferred Reporting Items for Systematic Reviews and Meta-Analysis”—PRISMA [44], the protocol was carried out in the months of July and August 2020 and consisted in four stages (Figure 1): (1) Identification: The first author (A.d.l.R.R) found 1418 studies in the four digital databases; (2) Screening: The first author (A.d.l.R.R) eliminated the duplicate files (*n =* 167) and excluded those considered not relevant through a previous reading of the title, abstract and keywords (*n =* 1040). Furthermore, the first author (A.d.l.R.R), jointly with the second (J.L.C.), third (D.M.L.) and fourth author (A.L.), rejected the studies linked to the RAE according to the exclusion criteria through a full-text reading (*n =* 188); (3) Eligibility: The first (A.d.l.R.R), second (J.L.C.) and fourth author (A.L.) eliminated full-text studies from the selection process by the type of publication (*n =* 4) and systematic review (*n =* 8); (4) Inclusion: The remaining studies (*n =* 9) based on the relationship between the RAE and the competition performance in basketball were finally considered.

### 2.4. Data Extraction and Management

A standardized form was used to extract data from the studies included in the review for assessment study quality and scientific evidence. Thus, information about (A) “year of publication”, (B) “author/s”, (C) “sample characteristics” (number of players, gender, age and age group), (D) “sport context” (competition category and competition level), (E) “grouping method” (based on birthdate distribution: Quartile [Q], semester [S] or quartile and semester [Q + S]), (F) “competition performance measurement indicators” (type of result: Individual and/or collective; performance production period: Short term and/or long term), and (G) the relationship between the RAE and the competition performance (impact of the RAE, impact of the RAE Reversal or lack of impact) were collected.

### 2.5. Data Synthesis

Due to the heterogeneity and variability of the extracted data, the meta-analysis was not considered appropriate. Instead, in order to conduct an in-depth analysis of the impact on competition performance the sample was distributed into different subcategories. From each study, the data connected to the samples (“n”), the players (“n” and “%”) and the association between the RAE and competition performance (“n” and “%”) were provided.

- Sample Characteristics

Based on the sample characteristics (C), basketball players were grouped according to (C1) “gender”: Men and women; (C2) “age group”: Adolescence (13–14 years); post-adolescence (15–19 years); adults (>19 years) [45,46,47].

- Sport Context

With regard to the sport context (D), the basketball players were allocated according to (D1) “competition category”: U-14, U-15, U-16, U-17, U-18, U-19, U-20, U-21, U-22, or over 22 years-old; (D2) “competition level”: Local/regional, national or international.

- Grouping Method

In relation to the sample distribution and grouping method (E), players were categorized according to the birthdate and official cut-off date approved by the corresponding sport federation/organisation. Thus, the basketball players were divided, into annual competition cycle, by: (E1) ‘Semesters’. Semester 1 (S1) and Semester 2 (S2); (E2) ‘Quartiles’. Quartile 1 (Q1), Quartile 2 (Q2), Quartile 3 (Q3), Quartile 4 (Q4).

- Sport Performance Indicators

With regard to the competition performance (F), the scientific evidence was registered according to (F1) “type of result” (individual or collective); (F2) “performance production period” (short term associated with statistical parameters extracted from short tournaments or regular seasons (offensive: Games played, minutes played, points scored, point average, % effectiveness, % effectiveness 2 points, % effectiveness 3 points, free throws, assists, offensive rebounds, turnovers, personal faults received; defensive: Personal faults committed, defensive rebounds, steals, blocked shots; overall player rating: Performance index rating—PIR), or long term referred to attainments achieved throughout the sport career/sport period based on statistical parameters accumulated individually and/or collectively). Combining both measurement criteria, the sample was categorized into four groups: Short-term individual performance (personal statistics in competition); short-term collective performance (final team classification in competition); long-term individual performance (success throughout the sport career); long-term collective performance (team rankings and maintenance period).

- Relationship between RAE and Competition Performance

The samples were grouped by the influence of the relative age effect (RAE) on competition performance (G). Thus, the basketball players were included in one of the following groups: (G1) impact of the RAE on competition performance; (G2) impact of the RAE Reversal on competition performance; (G3) no association between the RAE/RAE Reversal and competition performance.

### 2.6. Study Quality Assessment

An adapted version “Strengthening the Reporting of Observational Studies in Epidemiology—STROBE” checklist [47,48] was employed to determine the study quality. The checklist was made up of 20 items grouped into six categories corresponding to the different sections of the study: “Title-Abstract” (item 1), “Introduction” (items 2–3), “Methods” (items 4–10), “Results” (items 11–15), “Discussion” (items 16–19) and “Funding” (item 20). A score of “0” was awarded to the items with lack of information, and “1” to the items accurately described. The total score resulting from the addition of the item values, considering the following levels: “Very low quality” (0–4 points); “low quality” (5–8 points); “medium quality” (9–12 points); “high quality” (13–16 points); and “very high quality” (17–20 points). Two independent reviewers (A.d.l.R.R. and J.L.C.) conducted study quality assessment. Rating disagreements were resolved by A.L. and inter-rater reliability calculated.

## 3. Results

### 3.1. Synthesis of Findings (Qualitative Analysis)

#### 3.1.1. Sample Characteristics and Sport Context

Scientific evidence from the descriptive analysis of the systematic review studies is presented in Table 1. The format and design, including (A) the year of publication, (B) the title and author, (C) the sample characteristics (overall number, gender, age), (D) the characteristics of the sport context (competition category, competition level and competition period), (E) the grouping method (quartiles and/or semesters), and (G) the impact of the birthdate on competition performance (relative age effect (denoted as “RAE”), relative age effect reversal (denoted as “RAE R”) or no effect (denoted as “No RAE”)), are provided. The studies are arranged chronologically to favour the interpretation and longitudinal evaluation of the findings.

#### 3.1.2. Sample Distribution

Considering the set of basketball players in whom the RAE was detected, a summary based on player characteristics and sport context is included in Table 2. The details with regard to the sample characteristics (C) are as follows:

(1) Gender. Relatively older basketball players were over-represented in 74% of the samples (*n* = 9822 players). Among these samples, the number of basketball players affected by the RAE was higher in the male category (*n* = 5415) than in the female category (*n* = 4407). A portion of the players (26%) were not affected by the RAE.
(2) Age group. We identified the RAE, with a higher frequency, in the “post-adolescence” group (15–19 years old) where 6894 basketball players were registered (52%). The ratio between the number of players affected by the RAE and those who were not influenced was greater as the player’s chronological age increased (adolescence, 1.5:1; post-adolescence, 3.5:1; adult, 3.7:1). There was a lack of RAE in seven samples. No cases of RAE reversal were found in any “gender” or “age group” subcategory.

The details with regard to the sport context (D) are as follows:

(1) Competition category. In the players’ formative ages, there was a prevalence of the samples in which the selection process to participate in official competitions was biased in favour of relatively older players (U-14, *n* = 4; U-15, *n* = 2; U-16, *n* = 2; U-17, *n* = 4; U-18, *n* = 6; U-19, *n* = 3; U-20, *n* = 3; U-21, *n* = 1; U-22, *n* = 1). Moreover, there was no RAE impact in the U-15, U-17, U-18, U-20 and U-22 categories. By contrast, there was a 3.8:1 ratio in favour of a lack of RAE, associated with the number of players, in over 22-years-old category.
(2) Competition level. There was a notable RAE presence in the samples at national and international competition levels (73%), including 9760 basketball players, while in local/regional competitions, the impact of RAE was minimal (*n* = 62). With regard to the relationship between the number of players affected by RAE and unaffected by the RAE, the ratio was higher in international competitions (4.4:1) than in national competitions (2.2:1). No cases of RAE reversal were found in any “competition category” or “competition level” subcategory.

#### 3.1.3. Association between the RAE and Competition Performance 

Table 3 shows the relationship between the RAE and competition performance in basketball (G) based on performance indicators (F).

Considering the measurement indicators in relation to (F1) the type of result and (F2) the performance production period, Table 4 shows the relationship between RAE and competition performance (*n =* 16,947). We found: (a) There were more cases with an association between the player’s birthdate and competition performance, in cases where the RAE was detected (57% measurements). No relationship between the RAE and competition performance was found in 43% of measurements; (b) there was an association between RAE and performance, to a greater extent, in short-term collective performance measurements (14 samples; 39% of measurements); and (c) no impact of the RAE on individual and/or collective long-term competition performance was found.

Based on the in-depth analysis, considering the sample characteristics (C), we found the following:

(1) Gender. The RAE showed a higher impact on performance in men (*n* = 6944) than in women (*n* = 2823). In men, the short-term collective performance was most affected by the RAE (25% measurements), while the RAE had no impact on competition performance in women (28% measurements).
(2) Age group. The greatest influence of the RAE on competition performance was occurred in the “post-adolescence” development stage, (33% of measurements). The most affected performance was the short-term collective performance (4539 measurements). However, this stage also showed that the RAE had a very low impact on performance (38% of measurements). In the other development stages, the influence of the RAE was greater, mainly, on the short-term collective performance measurements (“adolescence”, 9%; “adult”, 5%).

When we examined the sport context (D), we found the following:

(1) Competition category. A transition process was observed as basketball players participated from the youngest categories (U14-U18), where a greater influence of the RAE on competition performance was identified (7502 measurements; 44%), to the higher formative categories (U19-U22), in which the RAE had much less impact on competition performance (3102 measurements; 18%). In basketball players over 22 years-old, there were only 144 measurements (1%), in which there was an impact of the RAE on short-term collective performance.
(2) Competition level. At the national level, there were more measurements in which the RAE influenced performance (40%), especially the short-term collective performance (32%). However, at the international level, a balance was observed between the cases in which the RAE showed an impact on performance (*n* = 2965) and those in which no influence was identified (*n* = 3020). At the local/regional level, there were only 62 measurements, in which there was an impact of the RAE on short-term individual performance.

#### 3.1.4. Correlation between the RAE and Individual Short-Term Statistical Performance Parameters

The impact of the RAE on the individual statistical performance parameters used in basketball are presented in Table 5. Based on the sample characteristics (C):

(1) Gender. All the statistics were influenced by the RAE in men, except “assists” and “steals”. In women, the RAE exerted a greater impact on “assists/min” and “steals/min”.
(2) Age group. The RAE impacted all statistics in the post-adolescent stage. In adolescence, the RAE slightly influenced the “points scored/min” and the “performance index rating (PIR)/min”. No impact of the RAE was found in the adult stage.

Considering the sport context (D), we found the following:

(1) Competition category. The RAE impacted all the statistical parameters, to a greater or lesser extent, in the U14-U18 and U19-U22 categories, except for the “blocked shots” in the U19-U22 categories. No impact of the RAE was detected in the over 22-years-old categories.
(2) Competition level. At the national level, the RAE had a greater impact on the “points scored”, “the blocked shots” and “PIR”, while in international competitions, the RAE influence was greater on “minutes played”, “% of effectiveness”, “assists/min” and “rebounds”.

The impact of the RAE reversal was found on statistical parameters, although the relationship was not significant and/or tangible to identify better overall competition performance in relatively young players. In the short term, the impact of the RAE reversal was detected mainly in the “% of effectiveness” and “2 points % of effectiveness” statistics in post-adolescent women (U-19 category) at the international level. In the long term, the RAE reversal mainly impacted the performance indicator “games played” throughout a sport career in national competitions and categories over the age of 22.

### 3.2. Study Selection and Assessment (Quality Analysis)

The quality analysis (“RAE-Performance Strengthening the Reporting of Observational Studies in Epidemiology (STROBE)” checklist) yielded the following results (Table 6): (a) The quality scores ranged from 11 to 19; (b) the average score was 15.22 points; c) of the nine included studies, two (22%) were considered “medium quality” (9–12 points); three (33%) were categorized as “high quality” (13–16 points); and four (45%) were considered “very high quality” (17–20 points).

The highest scores were located in the “Methods” (83%), “Discussion” (81%) and “Introduction” (78%) sections. Among the highest quality studies, we considered items no. 3 (“Objectives—State specific objectives and/or any pre-specified hypothesis”), no. 8 (“Data Source—Procedure for determining performance measurement”), no. 11 (“Descriptive Results—The number (absolute frequency) or percentage (relative frequency) of participants found in each grouping category and subcategory”) and no. 16 (“Key Results—A summary of key results with reference to study objectives”) to be complete (100%). By contrast, the most commonly absent or incomplete item (0 points) was no. 14 (“Main results—A measure of effect size” (67%)). The lowest scores were in the “Abstract” section (44%).

## 4. Discussion

**Figure 2:**
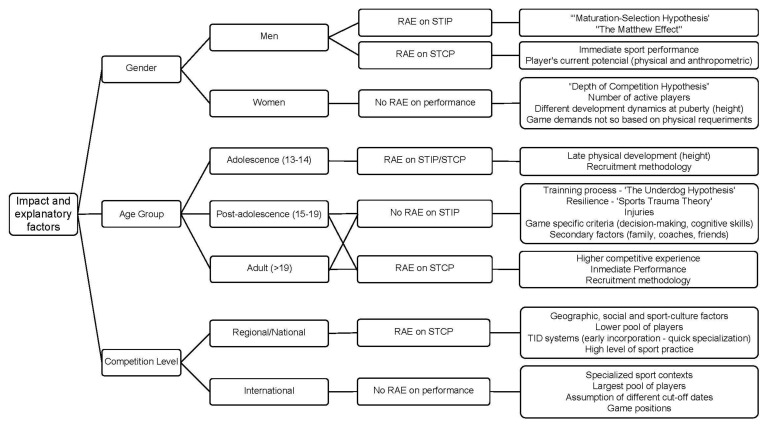
Summary of impact and explanatory factors of the impact/non-impact of the relative age effect (RAE) on competition performance in basketball. Notes: “RAE” = relative age effect; “STIP” = short-term individual performance; “STCP” = short-term collective performance; “TID” = talent identification and development.

The purpose of this study was to analyze whether there is a difference in competition performance between relatively older players (born at the beginning of the same constituent year) and relatively young players (born at the end of the same constituent year). According to the extracted results, we found that there was (a) an impact of the RAE on competition performance in 55.86% of the measurements and (b) a greater influence of the RAE on short-term collective performance (54.16% of measurements). Furthermore, the sample characteristics and the sport context were modifying factors of the impact of the RAE and its influence on competition performance in basketball (Figure 2).

### 4.1. RAE and Competition Performance by Gender

With regard to “gender”, the RAE had a greater impact on competition performance in men, especially on collective short-term performance; in women, the results mainly showed a lack of influence of the RAE on competition performance, both individually and collectively.

In men’s basketball, researchers have reported similar results [7,36,37,49,50]. This fact can be explained by the player selection process and the performance production period. Teams competing in a short-length tournament (i.e.,: World Championships or European Championships) or in a season (leagues) expect to reach immediate performance supported by the players’ current potential [42]. Thus, coaches tend to choose players with a high level of easily identifiable skills (physical and anthropometric), favouring early developers [51]. Therefore, this player selection model gives rise to team rosters that are composed of a majority of relatively older players due to greater maturational development [7,36,49], discriminating against other players, based on their birthdate, who could be considered potentially “talented” [52].

Furthermore, basketball teams made up mainly of relatively older players performed better, that is, they achieved higher positions in the competition [7,49]. Accordingly, it seems logical to think that men’s basketball is affected by the RAE due to biological factors, such as “YAPHV” [27] or height [36]. The “maturation-selection hypothesis” [25,53] would acquire special relevance in the player selection process, having an impact on individual short-term performance and, therefore, on the collective short-term competition performance [37]. Another possible explanation in this regard would be based on the fact that relatively older basketball players, who belong to national teams or clubs located in contexts where basketball is a popular sport, could benefit from better training conditions and higher competition levels [22]. Therefore, “when” and “where” would also become decisive factors to achieve sporting success. This phenomenon is called “the Matthew effect” [54]. Esteva et al. [55] provided a clear example of this theory in basketball. The social factors associated with the sport development process in Spain produced a series of advantages for relatively older players who, later, maintained positions at top competition levels.

On the other hand, in women’s basketball, even with an overrepresentation of relatively older players, the RAE could not be considered a differential factor of individual and collective competition performance between players born at the beginning of the year and those born at the end of the same year. Factors such as the “depth of the competition” [56], the number of active participants [47] or the different maturational process [2] could explain the lower magnitude of the RAE. Furthermore, different developmental dynamics at puberty [57], an accelerated stabilization of conditional-biological differences [36] and different game demands based, to a lesser extent, on physical, tactical and performance requirements [58,59,60], may have caused the RAE to have no impact on competition performance in women’s basketball.

### 4.2. RAE and Competition Performance among the Age Group: Competition Category

With regard to the competition category, the present findings demonstrated the highest magnitude of the RAE in the adolescence stages and youth categories, according to other reviews [25,47,61] and, furthermore, a greater degree of impact on competition performance. One of the significant factors that explains this difference among players with different relative ages, especially in the early stages of the sport transition process, would be “height” [36]. This physical parameter is mainly influenced by chronological age and “YAPHV”. Thus, this fact could determine the presence of taller players in elite teams [62], causing a starting disadvantage in the performance (participation and success) of relatively young players [27]. However, this criterion does not appear in all player positions. While forwards and pivots depend, to a greater extent, on height to obtain greater competition performance, relatively older guards could base their sporting success on other factors such as a greater amount of training, experiencing more competitive situations, or acquiring performance skills more quickly than the relatively young players [39]. Nevertheless, the paradigm of the player selection process for the different playing positions through classic functionalities or specific characteristics is being replaced, especially in competitions such as the NBA or the NCAA, by a process based on the tactical conception of the coach/staff [63]. Thus, the selection of players appears to be made according to the technical–tactical roles based on statistical data (internal variability) to the detriment of the traditional assignment of the playing position according to physical and anthropometric capacities (external variability) [41].

However, limiting factors, such as height, tend to disappear as the player’s growth process ends [7], tending to equalize as high-performance [64,65]. This trend is more notable in the exterior players (guards and forwards) than in the interior players (pivots), in whom anthropometric parameters continue to have special relevance for selection and to achieve high performance [66]. This fact could be due to different factors: (a) Training process: To overcome physical and anthropometric limitations, relatively young players would develop superior technical and tactical skills. Thus, in adulthood, when maturational differences diminish or disappear, these skills would allow them to perform as well or better than those born at the beginning of the year [25]. Relatively young players who overcome this fact and other types of difficulties (less attention from coaches, deselection from elite teams, less psychological maturity, etc.) is what is known as the “Underdog Effect” [35]. (b) Resilience: A great effort in the learning process [67], exposure to adverse developmental experiences [32] and more stressful training situations under pressure experienced in youth categories [68], even “traumas” [69], could explain why relatively young players achieve superior performance with no differences with regard to relatively older players; (c) Injuries: Less participation in competitions in formative categories would mean a lower injury rate and, therefore, fewer sport drop-outs by relatively young players, allowing them to reach high levels of performance [70]; (d) secondary factors: Family, coaches, training conditions, genetics, etc. could help turn players born at the end of the year into experts and thus match their performance with the relatively older players [71].

Although, the influence of the RAE on short-term individual performance was not marked, it was observed that the teams who performed better (final team position) in the U-14/U-19 and over 22-years-old categories were mainly made up of relatively older players. After the initial stages, it seems that coaches, especially at high levels of competition, tend to select those players who can perform immediately [72]. In this way, the recruiting system is biased in favour of relatively older players who enjoy more playing time and, therefore, have the possibility of producing more than the relatively young ones [7,73]. If we understand the collective competition performance (final team position) as an addition to individual performances (statistics parameters), it seems that a team with more basketball players born at the beginning of the year has better chances of winning. Furthermore, increased competitive experience due to an early identification and detection of talent would allow the relatively older players to have better training conditions and, therefore, lead to improved individual and collective performance [7,22,39]. However, this system based on short-term performance indicators could leave out specific criteria of the game such as leadership, cognitive skills or decision-making [74].

### 4.3. RAE and Competition Performance by Competition Level

Although the magnitude of the RAE in basketball according to the “competition level” has appeared inconsistently in the scientific literature [34,49,75,76], one of the explanations for this phenomenon would be based on the pool of players available for selection. While in national competitions the recruitment of players is limited by geographic, social or sport-culture-related factors [77], at the international level these limitations disappear. Thus, decision-making regarding the selection process is more associated with technical-tactical factors [78]. Another explanation is the gap between the start of official competitions at the national level and the international level leading to, in many cases, the maturational process being in its final stage or having already ended. Those filters would cause the RAE and its impact on performance to have less relevance in specialized sport contexts [79].

With regard to national competitions, a sport transition focused on the result and not on the process, based on an early incorporation, a quick specialization in the sport, a high volume of specific practice and a high domain of specific skills, could determine higher levels of short-term individual and collective performance [80]. Several studies carried out in national contexts support this research line [7,23,37,81,82], identifying how the teams that achieved a better classification in their respective competitions were mainly made up of relatively older players.

At the international level, previous literature has shown mixed results. The impact of the RAE on short-term competition performance, in addition to the pool of selectable players, could be modulated by a determining factor in team sports: The playing position [73,75,83]. The common conclusion of these studies was associated with the “maturation–selection hypothesis” [25]. Thus, the positions that demanded greater physical and anthropometric requirements were occupied by relatively older players, while those positions that demanded other factors less associated with conditional capacity were occupied by relatively young players. Therefore, depending on the position, the RAE would have more or less impact on competition performance in basketball [7,39,50].

### 4.4. RAE and Short-Term Competition Performance (Statistics)

While the RAE had a greater influence on the short-term collective competition performance, certain individual statistical parameters were also affected. The RAE exerted a greater impact on competitive contexts (men, post-adolescence group, U14-U18 categories) where the physical and anthropometric component has greater importance in the game [7,36]. Boys who reach puberty early tend to develop higher anthropometric values of height (approximately 20 cm) and vertical jump agility (approximately 12 cm) [84]. Therefore, statistics as decisive as the points scored or minutes played in a match clearly favour the type of players [85] who correspond, to a greater extent, to those born at the beginning of the year. This combination between early maturation and the effect of birthdate (relatively older players) represents a clear individual performance advantage in basketball, which seems to lead to a higher collective performance in teams made up by these kind of players [37].

With regard to the competition level, the relationship between the RAE and statistical performance parameters is not clear. However, if we considered a global performance indicator, such as the “PIR”, the RAE becomes more evident in national competitions. In these contexts, in which there is greater heterogeneity in terms of player profiles, physical and anthropometric factors (arm span, hand length, “APHV” and height) are highly influential towards performance (“PIR”) [37].

### 4.5. Study Quality Assessment

When were categorized studies based on “quality” according to the adapted version of the Strengthening the Reporting of Observational Studies in Epidemiology (STROBE) checklist [47,48], we drew the following conclusions: (a) The studies that yielded better quality scores (17–20 points) were associated with the analysis and evaluation of, almost exclusively, individual short-term performance indicators in the U14-U18 and U19-U22 categories. Moreover, these studies provided a high number of indicators linked to game statistical parameters, which demonstrates the degree of thoroughness and precision of the results. Thus, the main common finding identified in “very high quality” studies (17–20 points) was that the RAE did not impact individual competition performance; (b) the lowest quality score (11 points) was associated with a lack of information in the “Results” section and not providing a solid correlation between the RAE and the competition performance (collective performance based on final team position).

However, it could not be confirmed that a high/low study quality score was linked to a specific trend in terms of the impact of the RAE on competition performance. These findings highlight the need to provide complete data, especially in the “Methods” and “Results” sections, to be able to carry out more complete analyses. The study quality assessment can be a useful procedure in subsequent studies to examine the strengths and weaknesses of the scientific investigations and provide information on the improvement points of the studies.

### 4.6. Limitations

First, there may have been a possible contamination of the data linked to the relative age of the players due to the non-specification of the birth quarter according to the year, that is, not separating players from the first and second year of the same competitive cycle. Second, there was an insufficient number of samples of basketball players in relation to the local/regional contexts (to be considered a competition level) that can be analyzed in a valid way. Third, no collective long-term performance measurement was registered and only one study provided data on long-term individual performance. Thus, it is not possible to know exactly how the RAE could affect competition performance during the sport career of basketball players and, therefore, the collective results of their corresponding teams. Fourth, the extraction of results and conclusions associated with the purpose of the systematic review may not have been as accurate as possible due to the great sampling and methodological diversity of the studies. Fifth, some game actions (intangibles), which are particularly valued by coaches, were not included in the statistics and, therefore, have not been considered as performance measurements indicators in this study. Sixth, there was no quantitative review of the results through the meta-analysis technique that allowed drawing conclusions linked to the objective of establishing common points between studies.

### 4.7. Practical Applications

Player selection processes should pay attention to the subsequent consequences of the RAE in terms of competition performance to ensure fairness and equal opportunities. Therefore, it would be convenient for coaches, stakeholders and talent scouts to implement the following strategies: (a) Analyse the sport performance of a player from a long-term approach that does not seek immediate results; (b) organise official competitions according to the maturational level of the players, without taking into account age ranges; (c) design player detection and selection protocols and models that are not focused on physical and anthropometric patterns but consider psychosocial factors, emotional intelligence and cognitive skills; (d) implement rules in the player selection and participation processes (establishment of a maximum average age per team, limit a maximum number of relatively older/younger players, organize competition categories with less variability in terms of the players’ birthdate); and (e) build a non-exclusive talent development system (“inside” or “outside”) so that non-selected players remain in the system, allowing them to continue developing athletically.

### 4.8. Future Research

With the aim of obtaining as much information as possible in relation to player selection processes, research could be expanded to other team sports with sufficient scientific evidence (football or ice hockey) or even to individual sports, after normalizing the performance results. Furthermore, there should be a focused investigation into the impact of the RAE on competition performance in team sports considering some more individual constraints (handedness), task constraints (laterality), and environmental constraints (family or coach influence). At the statistical level, future studies should carry out a meta-analysis that presents more powerful conclusions with regard to the relationship between the RAE and competition performance. Finally, progress could be made in the design of competitions that try to overcome or reduce the RAE and analyze what kind of consequences it has, both in short-term and long-term performance. In this way, research from the descriptive and theoretical world (research through sport) could be combined with the experimental and practical world (research for sport).

## 5. Conclusions

The RAE had an impact on competition performance in basketball. The results highlighted the impact of the RAE on short-term collective performance, that is, with regard to final team classification. Moreover, all statistical parameters (short-term individual performance) were affected, to a greater extent, in men and the U14-U18 categories. Conversely, the RAE had little impact on competition performance in women and at the international competition level, considered separately. Notably, we found no studies in which the relationship between the RAE and collective long-term competition performance were evaluated. Only one study examined long-term individual performance, and the authors detected that the RAE had no impact. Furthermore, no sample was affected by RAE reversal.

With regard to the modifying factors of the RAE on performance in basketball (sample characteristics and sport context), we determined the following:

(1) There is a greater impact of the RAE on the short term individual and collective performance in male basketball players than in female players.
(2) There is a decrease in the influence of the RAE on short-term collective performance as the basketball player evolves towards the top or professional competitive levels.
(3) There is a higher impact of the RAE on performance, especially short term collective performance in national contexts compared with international sport contexts.
(4) Short-term individual performance (official statistics) is affected to a greater extent in men aged 14–19 years old, not appreciating a difference between the competition level.

## Figures and Tables

**Table 1:** Distribution of the sample according to the characteristics of the basketball players (n, age and gender), the sport context (competition category, competition level and competition period), grouping method (quartiles (Q) and/or semesters (S)) and its impact on the set of birthdates (relative age effect).

Author(s)	Sample Characteristics			Sport Context			Grouping Method	Relative Age Effect
	N	Age	Gender	Competition Category	Competition Level	Competition Period		
Torres-Unda et al. (2013)	46	13–14	M	U-14	ACB—Mini Cup of Spain (RL)	2010–2011	By semesters (S1–S2)	RAE
	16	13–14	M	U-14		2010–2011		RAE
García et al. (2014)	143	16–17	M	U-17	FIBA Basketball World Championship (IL)	2010	By quartiles (Q1–Q4)	RAE
	191	18–19	M	U-19		2011		RAE
	138	20–21	M	U-21		2005		No RAE
	144	16–17	F	U-17		2010		RAE
	194	18–19	F	U-19		2011		RAE
	144	20–21	F	U-21		2007		No RAE
Arrieta et al. (2016)	455	15–16	M	U-16	FIBA European Basketball Championship (IL)	2013	By quartiles (Q1–Q4)	RAE
	454	17–18	M	U-18		2013		RAE
	384	19–20	M	U-20		2013		RAE
	396	15–16	F	U-16		2013		RAE
	407	17–18	F	U-18		2013		RAE
	299	19–20	F	U-20		2013		No RAE
Steingröver et al. (2016)	407	-	M	>22	National Basketball Association-NBA (NL)	1980–1989	By quartiles (Q1–Q4)	No RAE
Torres-Unda et al. (2016)	72	13–14	M	U-14	ACB—Mini Cup of Spain (NL)	2010	By quartiles (Q1–Q4)	RAE
Rubajczyk et al. (2017)	1223	13–14	M	U-14	Polish Youth Basketball Championships (NL)	2013–2016	By quartiles (Q1–Q4) By semesters (S1–S2)	RAE
	927	15–16	M	U-16		2013–2016		RAE
	907	17–18	M	U-18		2013–2016		RAE
	792	19–20	M	U-20		2013–2016		RAE
	1228	13–14	F	U-14		2013–2016		RAE
	922	15–16	F	U-16		2013–2016		RAE
	900	17–18	F	U-18		2013–2016		RAE
	369	19–22	F	U-22		2013–2016		RAE
Zimmermann et al. (2017)	270	14–15	M	U-15	Brazilian Basketball Championship (NL)	2015–2016	By quartiles (Q1–Q4)	RAE
	260	14–15	F	U-15		2015–2016		No RAE
Ibañez et al. (2018)	334	17–18	M	U-18	Adidas Next Generation Tournament (NL)	2013–2014	By quartiles (Q1–Q4) By semesters (S1–S2)	RAE
	247	17–18	M	U-18		2014–2015		RAE
Vegara-Ferri et al. (2019)	192	16–17	M	U-17	FIBA Basketball World Championship (IL)	2016	By quartiles (Q1–Q4)	RAE
	192	18–19	M	U-19		2015		RAE
	144	-	M	>22	Rio de Janeiro 2016 Olympic Games (IL)	2016		No RAE
	180	16–17	F	U-17	FIBA Basketball World Championship (IL)	2016		RAE
	192	18–19	F	U-19		2015		RAE
	144	-	F	>22	Rio de Janeiro 2016 Olympic Games (IL)	2016		No RAE

Notes: N = absolute frequency of the sample; M = male; F = female; U-14 = under 14; U-16 = under 16; U-17 = under 17; U-18 = under 18; U-19 = under 19; U-20 = under 20; U-21 = under 21; U-22 = under 22; >22 = over 22 years-old; RL = regional level; NL = national level; IL = international level; Q1–Q4 = birth quarter; S1–S2 = birth semester; No RAE = no relative age effect; RAE = relative age effect; RAE R = relative age effect reversal. “-” = information does not provide.

**Table 2:** Summary of sample’s distribution (n and %) according to the relative age effect identified (RAE or No RAE) by characteristics of basketball players (gender and age group) and sport context (competition category and competition level).

		RAE		No RAE	
Category	Subgroup Category	Samples N	Basketball Players n(%)	Samples N	Basketball Players n(%)
**Sample Characteristics**	Gender				
	Male	15	5415(41)	5	2119(16)
	Female	12	4407(33)	2	1372(10)
	Age group				
	Adolescence (12–14)	6	1887(14)	1	1228(9)
	Post-adolescence (15–19)	17	6894(52)	4	1981(15)
	Adult (>19)	4	1041(8)	2	282(2)
**Sport Context**	Competition category				
	U-14	4	1357(10)	1	1228(9)
	U-15	2	530(4)	0	0(0)
	U-16	2	1318(10)	2	1382(11)
	U-17	4	659(5)	0	0(0)
	U-18	6	3249(25)	0	0(0)
	U-19	3	577(4)	1	192(1)
	U-20	3	1475(11)	0	0(0)
	U-21	1	144(1)	1	138(1)
	U-22	1	369(3)	0	0(0)
	>22	1	144(1)	2	551(4)
	Competition level				
	Local/Regional	2	62(1)	0	0(0)
	National	9	5715(43)	3	2562(19)
	International	16	4045(30)	4	929(7)

Notes: n = absolute frequency; % = relative frequency; U-14 = under 14; U-16 = under 16; U-17 = under 17; U-18 = under 18; U-19 = under 19; U-20 = under 20; U-21 = under 21; U-22 = under 22; >22 = over 22 years-old.

**Table 3:** Relationship between the relative age effect (RAE) and competition performance providing aim(s) of the study, performance indicators, main results and conclusion(s).

Author(s)	Aim(s) of the Study	Performance Indicators	Main Results (RAE-Performance)	Conclusion(s)
Torres-Unda et al. (2013)	Thus, in the present study, we compared the anthropometric, physiological, and motor characteristics of elite and non-elite young basketball players and the relationship between these parameters and performance	Individual statistics: point average (games played + points scored)	Relatively older players performed better according to “point average”, regardless of competition level (elite and non-elite) However, this relationship is only significative in “non-elite” group	Influence of RAE on short-term individual performance
García et al. (2014)	To check whether the relative age effect does exist in the World Basketball Championship U17, U19 and U21 male and female categories, to investigate if the relative age effect exists in the different specific positions and also try to find differences in height and in performance between players depending on their birthdate	Individual statistics: Games played; minutes played; converted field goals (% effectiveness); 2-point field goals (% effectiveness); 3-point field goals (% effectiveness); free goals scored (% effectiveness); def. rebounds; off. Rebounds; assistances; personal faults; stolen; recuperations; blocked; points; points per game	Relatively older players performed better on the following statistical parameters: 3-point % (male U-17); points per game (male U-19); assists and assists per game (female U-19) In contrast, relatively young players performed better on the following statistical parameters: 2-point % and free-throw % (female U-19) However, could be not affirmed, in general, that the competition performance in basketball, measured in statistical terms, was affected by the RAE	No relationship between RAE and short-term individual performance
Arrieta et al. (2016)	To analyze the presence of the RAE and the possible relation of relative age with performance in male and female European Youth Basketball Championships	Individual statistics: minutes, points, assists, steals, blocked shots, rebounds, personal fouls, missed shots, turnovers, personal, PIR Collective statistics: final team position	Relatively older players obtained higher individual performance indicators, in absolute and weighted terms, and collective performance according to final team position in competition than relatively young players in the U-20 category. The impact was less in U-16 and U-18 In women, the relationship between RAE and performance lost significance when the results were weighted for minutes played	Influence of RAE on short-term individual and collective performance (men) No relationship RAE -performance (women)
Steingröver et al. (2016)	To replicate previous findings on RAEs among NHL ice hockey players, NBA basketball players and NFL football players and in a second step to investigate the influence of relative age on career length in all three sports	Individual statistics throughout the sports career: Games played	Relatively young players played more games throughout their professional NBA career. However, it was no tangible relationship Considering the individual ranking, the relatively young NBA players with a medium/high individual ranking (positions 25th–75th), played more games than the relatively older players.	No relationship between RAE and long-term individual performance (NBA)
Torres-Unda et al. (2016)	To compare anthropometric, maturational, and physical performance variables regarding the performance of the teams in a championship. In addition, another objective was to explore the relationship between maturity-related parameters, anthropometric variables and physical performance variables of boys enrolled in elite basketball teams and the relationship between these parameters and their performance in basketball	Individual statistics: points per minute; points per game; index performance rating (PIR) and time played per game (min) Collective statistics: final team position	A relationship between relative age, when the player reached the maximum Peak Height Velocity (YAPHV), and performance was observed, in terms of points scored and performance index rating (PIR). This relationship decreased when the results were weighted by the min. An early maturation (YAPHV) and advanced maturity status was identified as key factors to reach the highest levels of performance. Relatively older players performed better than relatively young peers Relatively older players were overrepresented in those basketball teams that performed better in competition based on the final position	Influence of RAE on short-term individual and collective performance
Rubajczyk et al. (2017)	To identify the RAE in youth basketball games in Poland while taking into consideration the age, sex and the players’ match statistics. Additionally, the aim of this study is to determine whether differences in the body height of players are associated with the success of the team	Individual statistics: points per game; assists per game; rebounds per game; steals per game; blocks per game; turnovers per game; performance index rating (PIR) Collective statistics: Final team position	Relatively older players achieved higher individual performance parameters than relatively young players in U-14 men category. No impact of the RAE on competition performance was observed in the remaining male categories (U-16, U-18 and U-20) and in women Relatively older players (with higher height) scored more points per game than relatively young players in male and female U-14 category The teams with the worst classification in the men’s competitions showed roster made up mainly of players with a bigger height differential between the relatively older players (Q1) and the relatively young peers (Q4) than the teams that performed better (final position)	Influence of RAE on short-term individual performance (male U-14) No relationship RAE and short-term individual performance (male U-16, U-18, U-20 and female) Influence of RAE on short-term collective performance
Zimmermann et al. (2017)	Thus, the aim of the present study was to investigate RAE in U-15 athletes of the 2015 Brazilian Basketball Championship, analyzing possible differences between sexes, geographic region, competitive level and team performance.	Collective statistics: Final team position	The teams with the best classification (medalist), both men and women, showed roster made up mainly of relatively older players The teams with intermediaries and lowers positions in men competition showed roster made up mainly of relatively older player. However, the RAE was not identified for this kind of teams in women’s competition	Influence of RAE on short-term collective performance (women) No relationship RAE and short-term collective performance (men)
Ibañez et al. (2018)	(i) To examine the distribution of birth dates in competitive basketball in the U-18 category, differentiating by playing position and ii) to analyze the effect of the RAE on performance according to playing position using performance indicators	Individual statistics: points scored, tried and successful two- and three- point shots, tried and successful free throws, total rebounds, defensive and offensive rebounds, assists, steals, turnovers, blocks committed and received, dunks, personal fouls committed and received, performance index rating (PIR) and minutes played	Relatively older players, who occupied the “guard” position obtained higher competition performance in points scored, % effectiveness in 2-point shots and value of the performance index rating (PIR) than their relatively young peers Relatively older players, who occupied the “guard-forward” position performed better on blocks made than their relatively young peers Relatively older players who occupied the “center” position reached higher competition performance in points scored, 2-point shots and value of the performance index rating (PIR) than their relatively young peers	Influence of RAE on short-term individual performance
Vegara-Ferri et al. (2019)	The objective of this study is to analyze the presence of RAEs and their possible relationship with the performance of men’s and women’s basketball teams at the World Championship of Basketball under-17 (2016) and under-19 (2015) and the teams of men’s and women’s absolute basketball of the Olympic Games in Rio de Janeiro 2016. Thus, the underlying purpose of this research is to analyze the relationship between the distribution of the players’ birth dates and the position in the final classification of the championship, position on the field and height	Collective statistics: final team position	The teams with the best classification in U-17, U-19 and absolute categories (groups “A” and “B”), both men and women’s competition, showed roster made up mainly of relatively older players. Moreover, the teams with intermediate classification in men’s competition (group “C”). also showed a RAE The teams with worst classification in U-17, U-19 and absolute categories (group “D” in men and groups “C” and “D” in women’s competition) showed a balanced players distribution with no RAE	Influence of RAE on short-term collective performance

Notes: PIR (Performance Index Rating) = a statistical formula also used by the FIBA, the Euroleague and the Eurocup, as well as various European national domestic leagues to determine the player’s performance in match.

**Table 4:** Summary of samples (n) and performance measures—PM (n and [%]) within the relationship between the relative age effect (RAE) and competition performance by characteristics of athletes (gender and age group) and sport context (competition category and competition level).

**Gender**					
**Performance**		**Influence—RAE**		**No influence—RAE**	
		**Samples (n)**	**PM (n[%])**	**Samples (n)**	**PM (n[%])**
Men					
Performance (St)	IPI	8	2776(16)	2	1699(10)
	CPI	8	4168(25)	3	604(4)
Women					
Performance (St)	IPI	0	0(0)	6	3293(19)
	CPI	6	2823(17)	6	1584(9)
**Age Group**					
**Performance**		**Influence—RAE**		**No influence—RAE**	
		**Samples (n)**	**PM (n[%])**	**Samples (n)**	**PM (n[%])**
Adolescence (13–14 years)					
Performance (St)	IPI	4	1357(8)	0	0(0)
	CPI	3	1555(9)	1	270(2)
Post-adolescence (15–19 years)					
Performance (St)	IPI	3	1035(6)	7	4623(27)
	CPI	8	4539(27)	7	1774(11)
Adult (>19 years)					
Performance (St)	IPI	1	384(2)	1	369(2)
	CPI	3	897(5)	1	144(1)
**Competition Category**					
**Performance**		**Influence—RAE**		**No influence—RAE**	
		**Samples (n)**	**PM (n[%])**	**Samples (n)**	**PM (n[%])**
U14/U18 categories					
Performance (St)	IPI	7	2392(14)	4	3125(19)
	CPI	9	5110(30)	4	953(6)
U-19/U-22 categories					
Performance (St)	IPI	1	384(2)	4	1867(11)
	CPI	4	1737(10)	5	1235(7)
>22 categories					
Performance (St)	IPI	0	0(0)	0	0(0)
	CPI	1	144(1)	0	0(0)
**Competition Level**					
**Performance**		**Influence—RAE**		**No influence—RAE**	
		**Samples (n)**	**PM (n[%])**	**Samples (n)**	**PM (n[%])**
Local/Regional					
Performance (St)	IPI	2	62(0)	0	0(0)
	CPI	0	0(0)	0	0(0)
National					
Performance (St)	IPI	2	1295(8)	5	3890(23)
	CPI	8	5445(32)	1	270(2)
International					
Performance (St)	IPI	4	1419(8)	3	1102(7)
	CPI	6	1546(9)	8	1918(11)

Notes: n = absolute frequency; % = relative frequency; PM = performance measure; St = short term; U-14/U-18 = under 14/under 18; U-19/U-22 = under 19/under 22; >22 = over 22 years old; IPI = individual performance indicators; CPI = collective performance indicators.

**Table 5:** Impact of the relative age effect (RAE) on the offensive and defensive individual performance statistical parameters (number of basketball players) according to the sample characteristics (gender and age group) and the sport context (competition category and competition level).

Statistical Parameter	N	Sample Characteristics					Sport Context					
		Gender		Age Group			Competition Category			Competition Level		
		M	W	Adolescent	Post-Adolescent	Adult	U14-U18	U19-U22	>22	Regional	National	International
**Offensive Statistics**												
Games played	407_a_ *	✓				✓			✓		✓	
												
Minutes played	455 *	✓			✓		✓					✓
	384 *	✓			✓			✓				✓
Points scored	191 *	✓			✓			✓				✓
	455 #	✓			✓		✓					✓
	384 #	✓			✓			✓				✓
	72 #	✓		✓			✓				✓	
	1223 #	✓		✓			✓				✓	
	246 *	✓			✓		✓				✓	
	133 *	✓			✓		✓				✓	
Point Average	16 *	✓		✓			✓			✓		
% Effectiveness	194_a_ *		✓		✓			✓				✓
	455#	✓			✓		✓					✓
	384 #	✓			✓			✓				✓
% Effectiveness 2 pts	194_a_ *		✓		✓			✓				✓
	246 *	✓			✓		✓				✓	
% Effectiveness 3 pts	143 *	✓			✓		✓					✓
	133 *	✓			✓		✓				✓	
Assists	194 #		✓		✓			✓				✓
	384 #	✓			✓			✓				✓
	396 #		✓		✓		✓					✓
	407 #		✓		✓			✓				✓
	900 #		✓		✓		✓				✓	
Turnovers	384 *	✓			✓			✓				✓
	1223 #	✓		✓			✓				✓	
**Defensive Statistics**												
Rebounds	455 #	✓			✓		✓					✓
	384 *	✓			✓			✓				✓
	1223 #	✓		✓			✓				✓	
Personal Faults	384 *	✓			✓			✓				✓
	133 *	✓			✓		✓				✓	
Steals	396 #		✓		✓		✓					✓
	407 #		✓		✓			✓				✓
	1223 #	✓		✓			✓				✓	
	900 #		✓		✓		✓				✓	
Blocked Shots	1223 #	✓		✓			✓				✓	
	202 *	✓			✓		✓				✓	
	133 *	✓			✓		✓				✓	
**Overall Player Rating**												
PIR	455 #	✓			✓		✓					✓
	384 #	✓			✓			✓				✓
	72 #	✓		✓			✓				✓	
	1223 #	✓		✓			✓				✓	
	900 #		✓		✓		✓				✓	
	246 *	✓			✓		✓				✓	
	133 *	✓			✓		✓				✓	

Notes: “*N*” = number of basketball players; *U-14/U-18* = under 14/under 18; *U-19/U-22* = under 19/under 22; *>22* = over 22 years-old; *pts* = points; “a” = sample with a reversal RAE; “*” = absolute performance statistical parameters; “*#*” = absolute and/or weighted performance statistical parameters per time.

**Table 6:** Study quality assessment based on the adapted version of Strengthening the Reporting of Observational Studies in Epidemiology—“STROBE”.

Items “STROBE”	Torres-Unda et al. (2013)	García et al. (2014)	Arrieta et al. (2016)	Steingröver et al. (2016)	Torres-Unda et al. (2016)	Rubajczyk et al. (2017)	Zimmerman et al. (2017)	Ibañez et al. (2018)	Vegara-Ferri et al. (2019)
*1. Title/Abstract. Informative and balanced summary of what was done and what was found is provided	1	1	0	0	0	1	0	1	0
*2. Background. Scientific background and rationale for the investigation being reported is explained	1	1	0	1	1	1	1	1	0
*3. Objectives. State specific objectives and/or any pre-specified hypothesis	1	1	1	1	1	1	1	1	1
*4. Setting. Locations, and relevant dates for data collection are described: Study period, sport context and competition year(s)	1	1	1	0	1	1	1	1	1
*5. Participants. Give characteristics of the sample (overall number, age, gender)	1	1	0	0	1	1	1	1	1
*6. Participants. Procedure for selecting athletes (i.e., cut-off date) and the way grouping according study purposes (i.e., by Q) are described	1	0	1	1	0	1	1	1	1
*7. Data Source. Source and procedure for obtaining the birthdate and performance sample characteristics are described	0	1	1	1	0	1	1	1	1
*8. Data Source. Procedure for determining performance measurement is described	1	1	1	1	1	1	1	1	1
*9. Statistical Methods. Specific analytical methods used to examine subgroups and interactions (RAE—performance) are described	1	1	1	1	1	1	0	1	0
*10. Statistical Methods. How duplicates and missing data were addressed or incomplete data were handled (if applicable) is explained	1	0	0	1	1	1	1	1	1
*11. Descriptive Results. The number or percentage of participants found in each grouping category and subcategory are reported	1	1	1	1	1	1	1	1	1
*12. Main Results. Statistical estimate and precision (i.e., 95% IC) for each sample or subgroup is provided	0	1	1	1	0	1	1	1	0
*13. Main Results. Post-hoc comparisons (OR) between grouping category (i.e., Q1 vs. Q4) are provided	0	1	0	1	0	1	1	1	0
*14. Main Results. A measure of effect size is provided (i.e., Cramer’s V, phi coefficient, Cohen’s)	0	1	0	1	0	1	0	0	0
*15. Main Results. A coefficient of correlation between RAE and performance measures is provided	1	1	1	1	1	1	0	1	0
*16. Key Results. A summary of key results with reference to study objectives is provided	1	1	1	1	1	1	1	1	1
*17. Limitations. Limitations of the study, considering sources of potential bias or imprecision are discussed	1	0	0	1	1	1	0	1	0
*18. Interpretation. A cautious overall interpretation of results considering objectives and evidence is provided	1	1	0	1	1	1	1	1	1
*19. Generalizability. The generalizability of the study results to similar or other contexts is provided	1	1	1	1	0	0	1	1	1
*20. Funding. The funding source of the study is cited or the lack of funding, if applicable	0	1	1	1	1	1	0	1	0
SCORE	15	17	12	17	13	19	14	19	11

Notes: Title/Abstract = *1; Introduction = *2–*3; Methods = *4–*10; Results = *11–*15*; Discussion = *16–*19; Funding = *20; “0” = item with absence or lack of information; “1” = item with complete and explicit information.

## References

[1] Singer R.N., Janelle C.M. (1999). Determining sport expertise: From genes to supremes. Int. J. Sport Psychol..

[2] Konstantinos N.S., Elissavet R.N., Panagiotis G.M., Ioannis B.A., Konstantinos B.D. (2018). Performance indicators and competition ranking in women’s and men’s world. J. Phys. Educ. Sport.

[3] Hughes M.D., Bartlett R.M. (2002). The use of performance indicators in performance analysis. J. Sports Sci..

[4] Mateus N., Esteves P., Gonçalves B., Torres I., Gomez M.A., Arede J., Leite N. (2020). Clustering performance in the European basketball according to players’ characteristics and contextual variables. Int. J. Sports Sci. Coach..

[5] Dehesa R., Vaquera A., Gonçalves B., Mateus N., Gomez-Ruano M.A., Sampaio J. (2019). Key Game indicators in NBA players’ performance profiles. Kinesiology.

[6] Zhang S., Lorenzo A., Gómez M.A., Mateus N., Gonçalves B., Sampaio J. (2018). Clustering performances in the NBA according to players’ anthropometric attributes and playing experience. J. Sports Sci..

[7] Arrieta H., Torres-Unda J., Gil S.M., Irazusta J. (2016). Relative age effect and performance in the U16, U18 and U20 European basketball championships. J. Sports Sci..

[8] Lorenzo J., Lorenzo A., Conte D., Giménez M. (2019). Long-term analysis of elite basketball players’ game-related statistics throughout their careers. Front. Psychol..

[9] Ortega-Toro E., Bernal-Polo J., Gómez-Ruano M. (2019). Relación entre edad y criterios de rendimiento y participación en jugadores de baloncesto de alto rendimiento. Rev. Psicol. Deport..

[10] García J., Ibáñez S., De Santos R.M., Leite N., Sampaio J. (2013). Identifying basketball performance indicators in regular season and playoff games. J. Hum. Kinet..

[11] Lorenzo A., Gómez M.A., Ortega E., Ibáñez S., Sampaio J. (2010). Game related statistics which discriminate between winning and losing under-16 male basketball games. J. Sport. Sci. Med..

[12] Ibáñez S., Sampaio J., Feu-Molina S., Lorenzo A., Gomez M.A., Ortega-Toro E. (2008). Basketball game-related statistics that discriminate between teams’ season-long success. Eur. J. Sport Sci..

[13] Gómez-Ruano M., Ibáñez S., Parejo I., Furley P. (2017). The use of classification and regression tree when classifying winning and losing basketball teams. Kinesiology.

[14] Musch J., Grondin S. (2001). Unequal competition as an impediment to personal development: A review of the relative age effect in sport. Dev. Rev..

[15] De la Rubia A., Lorenzo-Calvo J., Lorenzo A. (2020). Does the relative age effect influence short-term performance and sport career in team sports? A qualitative systematic review. Front. Psychol..

[16] Barnsley R., Thompson A., Barnsley P. (1985). Hockey success and birthdate: The relative age effect. J. Can. Assoc. Health Phys. Educ. Recreat..

[17] Vaeyens R., Philippaerts R.M., Malina R.M. (2005). The relative age effect in soccer: A match-related perspective. J. Sports Sci..

[18] Williams J.H. (2010). Relative age effect in youth soccer: Analysis of the FIFA U17 world cup competition. Scand. J. Med. Sci. Sport..

[19] Till K., Cobley S., Wattie N., O’Hara J., Cooke C., Chapman C. (2010). The prevalence, influential factors and mechanisms of relative age effects in UK rugby league. Scand. J. Med. Sci. Sport..

[20] Wattie N., Cobley S., Baker J. (2008). Towards a Unified Understanding of Relative Age Effects. J. Sports Sci..

[21] Steingröver C., Wattie N., Baker J., Helsen W.F., Schorer J. (2017). Geographical Variations in the Interaction of Relative Age Effects in Youth and Adult Elite Soccer. Front. Psychol..

[22] Hancock D.J., Adler A.L., Côté J. (2013). A proposed theoretical model to explain relative age effects in sport. Eur. J. Sport Sci..

[23] Yagüe J.M., de la Rubia A., Sánchez-Molina J., Maroto-Izquierdo S., Molinero O. (2018). The relative age effect in the 10 best leagues of male professional football of the union of european football associations (UEFA). J. Sport. Sci. Med..

[24] Helsen W.F., Van Winckel J., Williams A.M. (2005). The relative age effect in youth soccer across Europe. J. Sports Sci..

[25] Cobley S.P., Baker J., Wattie N., McKenna J. (2009). Annual age-grouping and athlete development. A meta-analytical review of relative age effects in sport. Sport. Med..

[26] Baker J., Schorer J., Cobley S. (2010). Relative age effects. An inevitable consequence of elite sport?. Sportwiss.

[27] Torres-Unda J., Zarrazquin I., Gil J., Ruiz F., Irazusta A., Kortajarena M., Seco J., Irazusta J. (2013). Anthropometric, physiological and maturational characteristics in selected elite and non-elite male adolescent basketball players. J. Sports Sci..

[28] McCarthy N., Collins D. (2014). Initial identification & selection bias versus the eventual confirmation of talent: Evidence for the benefits of a rocky road?. J. Sports Sci..

[29] Brustio P.R., Lupo C., Ungureanu A.N., Frati R., Rainoldi A., Boccia G. (2018). The relative age effect is larger in Italian soccer top-level youth categories and smaller in serie A. PLoS ONE.

[30] Gil S.M., Bidaurrazaga-Letona I., Martin-Garetxana I., Lekue J.A., Larruskain J. (2019). Does birth date influence career attainment in professional soccer?. Sci. Med. Footb..

[31] Fumarco L., Gibbs B.G., Jarvis J.A., Rossi G. (2017). The relative age effect reversal among the national hockey league elite. PLoS ONE.

[32] McCarthy N., Collins D., Court D. (2016). Start hard, finish better: Further evidence for the reversal of the RAE advantage. J. Sports Sci..

[33] Till K., Cobley S., Morley D., O’Hara J., Chapman C., Cooke C. (2016). The influence of age, playing position, anthropometry and fitness on career attainment outcomes in rugby league. J. Sports Sci..

[34] Werneck F.Z., Coelho E.F., de Oliveira H.Z., Ribeiro Júnior D.B., Almas S.P., de Lima J.R.P., Matta M.O., Figueiredo A.J. (2016). Relative age effect in olympic basketball athletes. Sci. Sport..

[35] Gibbs B.G., Jarvis J.A., Dufur M.J. (2012). The rise of the underdog? The relative age effect reversal among Canadian-born NHL hockey players: A reply to Nolan and Howell. Int. Rev. Sociol. Sport.

[36] Rubajczyk K., Świerzko K., Rokita A. (2017). Doubly disadvantaged? The relative age effect in Poland’s basketball players. J. Sport. Sci. Med..

[37] Torres-Unda J., Zarrazquin I., Gravina L., Zubero J., Seco J., Gil S.M., Gil J., Irazusta J. (2016). Basketball performance is related to maturity and relative age in elite adolescent players. J. Strength Cond. Res..

[38] Delorme N., Raspaud M. (2009). The relative age effect in young french basketball players: A study on the whole population. Scand. J. Med. Sci. Sport..

[39] Ibañez S.J., Mazo A., Nascimento J., Garcıa-Rubio J. (2018). The relative age effect in under-18 basketball: Effects on performance according to playing position. PLoS ONE.

[40] García-Rubio J., Courel-Ibáñez J., González-Espinosa S., Ibañez S.J. (2019). La especialización en baloncesto. Análisis de perfiles de rendimiento en función del puesto específico en etapas de formación. Rev. Psicol. Deport..

[41] Stefanescu C.A., Teodorescu S. (2018). Model analysis of the basketball player efficiency according to the new classification adapted to european basketball. Phys. Educ. Sport Kinetotherapy J..

[42] Bailey R., Collins D. (2013). The standard model of talent development and its discontents. Kinesiol. Rev..

[43] Swann C., Moran A., Piggott D. (2015). Defining elite athletes: Issues in the study of expert performance in sport psychology. Psychol. Sport Exerc..

[44] Liberati A., Altman D.G., Tetzlaff J., Mulrow C., Gøtzsche P.C., Ioannidis J.P.A., Clarke M., Devereaux P.J., Kleijnen J., Moher D. (2009). The PRISMA statement for reporting systematic reviews and meta-analyses of studies that evaluate health care interventions: Explanation and elaboration. PLoS Med..

[45] Baxter-Jones A.D. (1995). Growth and development of young athletes. Should competition levels be age related?. Sport. Med..

[46] World Health Organization (2015). Salud de La Madre, El Recién Nacido, Del Niño y Del Adolescente: Desarrollo En La Adolescencia.

[47] Smith K.L., Weir P.L., Till K., Romann M., Cobley S.P. (2018). Relative age effects across and within female sport contexts: A systematic review and meta-analysis. Sport. Med..

[48] Vandenbroucke J.P., von Elm E., Altman D.G., Gøtzsche P.C., Mulrow C.D., Pocock S.J., Poole C., Schlesselman J.J., Egger M. (2014). Strengthening the reporting of observational studies in epidemiology (STROBE): Explanation and elaboration. Int. J. Surg..

[49] Zimmermann de Oliveira H., Borges Ribeiro Junior D., Macedo Vianna J., Werneck F.Z. (2017). Relative age effect in Brazilian basketball championship: Under 15 players. Braz. J. Kineanthropometry Hum. Perform..

[50] Vegara-Ferri J.M., García-Mayor J., Pérez A.M., Cabezos H. (2019). Effect of relative age in the basketball world championships sub-17, sub-19 and olympic games of Brazil 2016. J. Sport Health Res..

[51] Johnston K., Wattie N., Schorer J., Baker J. (2018). Talent identification in sport: A systematic review. Sport. Med..

[52] Gastin P.B., Bennett G. (2014). Late maturers at a performance disadvantage to their more mature peers in junior australian football. J. Sports Sci..

[53] Helsen W.F., Starkes J.L., Van Winckel J. (1998). The influence of relative age on success and dropout in male soccer players. Am. J. Hum. Biol..

[54] Nolan J.E., Howell G. (2010). Hockey success and birth date: The relative age effect revisited. Int. Rev. Sociol. Sport.

[55] Esteva S., Drobnic F., Puigdellivol J., Serratosa L., Chamorro M. (2006). Date of birth and success in professional basketball. Apunt. L Esport.

[56] Baker J., Cobley S.P., Winckel V. (2009). Gender, depth of competition and relative age effects in team sports. Asian J. Exerc. Sport. Sci..

[57] Baptista F., Rebocho L.M., Cardadeiro G., Zymbal V., Rosati N. (2016). Sex and maturity-related differences in cortical bone at the distal radius and midshaft tibia evaluated by quantitative ultrasonography. Ultrasound Med. Biol..

[58] Sampaio J., Ibañez S.J., Feu-Molina S. (2004). Discriminative power of basketball game-related statistics by level of competition and sex. Percept. Mot. Skills.

[59] Gómez M.A., Lorenzo A., Ibañez S.J., Sampaio J. (2013). Ball possession effectiveness in men’s and women’s elite basketball according to situational variables in different game periods. J. Sports Sci..

[60] Leicht A., Gomez M.A., Woods C.T. (2017). Team performance indicators explain outcome during women’s basketball matches at the olympic games. Sports.

[61] Dixon J.C., Horton S., Chittle L., Baker J. (2020). Relative Age Effects in Sport. International Perspectives.

[62] Drinkwater E.J., Pyne D.B., Mckenna M.J. (2008). Design and interpretation of anthropometric and fitness testing of basketball players. Sport. Med..

[63] Bianchi F., Facchinetti T., Zuccolotto P. (2017). Role Revolution: Towards a new meaning of positions in basketball. Electron. J. Appl. Stat. Anal..

[64] Leite N., Borges J., Santos S., Sampaio J. (2013). The relative age effect in school and federative sport in basketball. Rev. Psicol. Deport..

[65] Manonelles P., Alvarez J., Coloma M., Sainz de Aja C., Corona-Virón P., Giménez-Salillas L. (2015). Edad cronológica como factor de elección de jugadores de las selecciones españolas de baloncesto de formación. Arch. Med. Deport..

[66] Ibáñez S.J., Santos J.A., García-Rubio J. (2015). Multifactorial analysis of free throw shooting in eliminatory basketball games. Int. J. Perform. Anal. Sport.

[67] Roberts S.J., Stott T. (2015). A new factor in UK students’ university attainment: The relative age effect reversal?. Qual. Assur. Educ..

[68] Andronikos G., Elumaro A.I., Westbury T., Martindale R.J.J. (2016). Relative age effect: Implications for effective practice. J. Sports Sci..

[69] Collins D.J., MacNamara A. (2017). Making champs and super-champs—Current views, contradictions and future directions. Front. Psychol..

[70] Wattie N., Cobley S., Macpherson A., Howard A., Montelpare W.J., Baker J. (2007). Injuries in Canadian youth ice hockey: The influence of relative age. Pediatrics.

[71] Baker J., Horton S. (2004). A Review of primary and secondary influences on sport expertise. High Abil. Stud..

[72] Simonton D.K. (2001). Talent development as a multidimensional, multiplicative, and dynamic process. Curr. Dir. Psychol. Sci..

[73] De la Rubia A., Bjørndal C.T., Sánchez-Molina J., Yagüe J.M., Lorenzo J., Maroto-Izquierdo S. (2020). The relationship between the relative age effect and performance among athletes in world handball championships. PLoS ONE.

[74] Hyllegard R., Radlo S.J., Early D. (2001). Attribution of athletic expertise by college coaches. Percept. Mot. Skills.

[75] García M.S., Aguilar Ó.G., Romero J.J.F., Lastra D.F., Oliveira G.E. (2014). Relative age effect in lower categories of international basketball. Int. Rev. Sociol. Sport.

[76] Steingröver C., Wattie N., Baker J., Schorer J. (2016). Does relative age affect career length in North American professional sports?. Sport Med. Open.

[77] Côté J., Macdonald D.J., Baker J., Abernethy B. (2006). When “where” is more important than “when”: Birthplace and birthdate effects on the achievement of sporting expertise. J. Sports Sci..

[78] Karcher C., Ahmaidi S., Buchheit M. (2014). Effect of birth date on playing time during international handball competitions with respect to playing positions. Kinesiology.

[79] Rees T., Hardy L., Güllich A., Abernethy B., Côté J., Woodman T., Montgomery H., Laing S., Warr C. (2016). The great British medalists project: A review of current knowledge on the development of the world’s dest sporting talent. Sport Med..

[80] Weissensteiner J., Abernethy B., Farrow D., Müller S. (2008). The development of anticipation: A cross-sectional examination of the practice experiences contributing to skill in cricket batting. J. Sport Exerc. Psychol..

[81] Augste C., Lames M. (2011). The relative age effect and success in German elite U-17 soccer teams. J. Sports Sci..

[82] Grossmann B., Lames M. (2013). Relative age effect (RAE) in football talents—The role of youth academies in transition to professional status in Germany. Int. J. Perform. Anal. Sport.

[83] Romann M., Fuchslocher J. (2011). Influence of the selection level, age and playing position on relative age effects in Swiss women’s soccer. Talent Dev. Excell..

[84] Jakovljevic S., Macura M., Radivoj M., Jankovic N., Pajic Z., Erculj F. (2016). Biological maturity status and motor performance in fourteen-year-old basketball players. Int. J. Morphol..

[85] Erčulj F., Štrumbelj E. (2015). Basketball shot types and shot success in different levels of competitive basketball. PLoS ONE.

